# Dyadic Psychosocial eHealth Interventions: Systematic Scoping Review

**DOI:** 10.2196/15509

**Published:** 2020-03-04

**Authors:** Kelly M Shaffer, Ashley Tigershtrom, Hoda Badr, Stephanie Benvengo, Marisol Hernandez, Lee M Ritterband

**Affiliations:** 1 Center for Behavioral Health and Technology University of Virginia School of Medicine Charlottesville, VA United States; 2 Memorial Sloan Kettering Cancer Center New York, NY United States; 3 Baylor College of Medicine Houston, TX United States; 4 CUNY School of Medicine/City College of New York New York, NY United States

**Keywords:** behavioral medicine, caregivers, couples therapy, dyads, eHealth, family therapy, internet interventions, psychosocial interventions, review

## Abstract

**Background:**

Dyadic psychosocial interventions have been found beneficial both for people coping with mental or physical health conditions as well as their family members and friends who provide them with support. Delivering these interventions via electronic health (eHealth) may help increase their scalability.

**Objective:**

This scoping review aimed to provide the first comprehensive overview of dyadic eHealth interventions for individuals of all ages affected by mental or physical illness and their family members or friends who support them. The goal was to understand how dyadic eHealth interventions have been used and to highlight areas of research needed to advance dyadic eHealth intervention development and dissemination.

**Methods:**

A comprehensive electronic literature search of PubMed, EMBASE, Cochrane, Cumulative Index to Nursing and Allied Health Literature, and PsycINFO was conducted for articles published in the English language through March 2019. Eligible records described a psychosocial eHealth intervention that intervened with both care recipients and their support person.

**Results:**

A total of 7113 records were reviewed of which 101 met eligibility criteria. There were 52 unique dyadic eHealth interventions identified, which were tested across 73 different trials. Of the unique interventions, 33 were conducted among dyads of children and their supporting parent, 1 was conducted with an adolescent-young adult care recipient population, and the remaining 18 were conducted among adult dyads. Interventions targeting pediatric dyads most commonly addressed a mental health condition (n=10); interventions targeting adult dyads most commonly addressed cancer (n=9). More than three-fourths of interventions (n=40) required some human support from research staff or clinicians. Most studies (n=64) specified one or more primary outcomes for care recipients, whereas less than one-fourth (n=22) specified primary outcomes for support persons. Where specified, primary outcomes were most commonly self-reported psychosocial or health factors for both care recipients (n=43) and support persons (n=18). Results of the dyadic eHealth intervention tended to be positive for care recipients, but evidence of effects for support persons was limited because of few studies specifying primary outcomes for supporters. Trials of dyadic eHealth interventions were most commonly randomized controlled trials (RCTs; n=44), and RCTs most commonly compared the dyadic eHealth intervention to usual care alone (n=22).

**Conclusions:**

This first comprehensive review of dyadic eHealth interventions demonstrates that there is substantial, diverse, and growing literature supporting this interventional approach. However, several significant gaps were identified. Few studies were designed to evaluate the unique effects of dyadic interventions relative to individual interventions. There was also limited assessment and reporting of outcomes for support persons, and there were no interventions meeting our eligibility criteria specifically targeting the needs of older adult dyads. Findings highlight areas of research opportunities for developing dyadic eHealth interventions for novel populations and for increasing access to dyadic care.

## Introduction

### Background

Psychosocial interventions provide evidence-based behavioral, cognitive, and emotion regulation strategies to address mental and physical health conditions. These conditions not only affect the person diagnosed with illness but also their close family members and friends, particularly those who support the clinical care of the person with illness. Dyadic interventions that intervene with both the care recipient and their support person together have been found clinically useful [1-5]; however, most have been designed to be delivered in-person, limiting their ultimate scalability and accessibility [6]. Delivering dyadic interventions via electronic health (eHealth; ie, using information communication technologies to facilitate health care provision) may increase their uptake by overcoming structural and financial access barriers. A broad review of the literature on dyadic eHealth interventions is needed to better understand how these interventions have been used and to identify where further research is needed.

### Illness Affects Dyads Together

It is estimated that 43.5 million Americans provide informal, unpaid care to one or more adults or children with serious health conditions [7]. Of these, it is estimated that about 33.3 million provide such care to adult recipients only, 3.7 million to child recipients only, and 6.5 million to both adult and child recipients. About 15% of people who provide care are not legally defined family members of the care recipient (eg, friend or neighbor [7]), and many individuals do not identify with the term *caregiver* (eg, [8]). As such, the term *support persons* is used for this scoping review to broadly capture individuals who provide emotional and practical support to help a care recipient. Together, the support person and the care recipient comprise a *dyad*.

Support from family members and friends to seriously ill individuals is critical to the sustainability of the US health care system [9], yet it can place significant strain on these support persons. Compared with the general population, support persons report worse diet, exercise, and sleep [10-12]; worse symptoms of depression and anxiety [13-15]; and premature physical health decline [16-18]. These mental and physical burdens from caregiving also ultimately affect the care recipients. Distressed support persons are more likely to exhibit harmful caregiving behavior [19] and less able to meet the practical and social-emotional needs of the care recipient [20,21]. When one dyad member is distressed, the other is more likely to become distressed as well [22-25]. Care recipients and their support persons experience illness together, and the success of one person’s ability to cope with illness stressors affects the other’s [26-28].

### Intervening With Dyads

Dyadic interventions may use a range of strategies, such as communication skills training, cognitive behavioral therapy, education, and problem-solving training. These interventions share the commonality of including both the care recipient and their support person together within a singular program of care. Dyadic psychosocial interventions have been found effective to improve targeted outcomes for both care recipients and support persons [1-5,29,30]. As coping is interdependent between care recipients and support persons, there is promise that dyadic interventions may deliver synergistic benefits—meaning the cumulative benefits to both individuals from a dyadic intervention is greater than the sum of benefits of intervening with each member individually. In practice, there has been limited empirical study of such effects [1,30].

Even if there are no such synergistic effects, dyadic interventions may be an attractive way to extend psychosocial care access to support persons. Interventions specifically addressing support persons’ informational and psychosocial needs have rarely been implemented in health care settings—common institutional barriers include competing clinical demands and lack of funding [31]. Support persons tend to underutilize available caregiver-directed programs, in part because of a reluctance to *put their needs ahead* of those of the care recipient [32,33]. Dyadic interventions may therefore be perceived as more justifiable by both health care systems and support persons: the care recipient is a target of care, and support persons receive care as well.

Despite the promise of dyadic interventions, there are significant logistical and financial barriers impeding their dissemination [6]. Common practical barriers like scheduling difficulties and limited time [32-36] are compounded when both care recipients’ and support persons’ schedules must align. In addition, support persons often have inadequate health care coverage [37], meaning that obtaining such care is likely to be cost-prohibitive for many dyads. Disseminating dyadic interventions via the internet and other technologies may lower the barrier to entry and increase their affordability [38]. The internet fills an important gap in meeting health information needs [39], and this is particularly true for support persons, who are more likely than noncaregivers to seek health information on the Web [40]. Internet- and technology-based approaches are also more scalable from a health systems perspective: reduced labor costs, as well as minimal ongoing program costs, suggest the long-term cost-efficacy of eHealth interventions relative to traditional face-to-face care [41].

### Purpose of This Review

The aims of this scoping review were twofold. The first aim was to provide the first summary of available evidence on dyadic eHealth interventions that provide behavioral treatment and/or psychosocial support to care recipients of all ages affected by any mental or physical illness together with their primary support persons. The second aim was to identify gaps in this knowledge base. To date, there has been one pioneering review of dyadic eHealth programs among cancer survivors and their family caregivers [29]. There have been many reviews demonstrating the efficacy and acceptability of eHealth interventions among diverse populations of patients (eg, [42-55]) and support persons (eg, [56-68]). All of these reviews have summarized literature within certain disease or age group populations, which silos the literature. In contrast, this review is intentionally broad, summarizing the literature across care recipient health conditions (eg, mental health, obesity, and cancer), population subgroups (eg, pediatric and adult care recipients), and intervention strategies (eg, communication training and cognitive behavioral therapy). Aims were well suited to a scoping review, which are ideal for reviewing a large and complex body of research that has not been previously reviewed [69,70]. The specific research questions that guided this review were as follows:

1. What populations have been targeted by dyadic eHealth interventions?
2. How have information communication technologies been used in dyadic eHealth interventions?
3. What approaches are used for intervening with both dyad members?

## Methods

A comprehensive electronic literature search for articles in the English language and for all available dates was conducted in the following databases: PubMed, EMBASE via the Elsevier platform, Cochrane via the Wiley platform, Cumulative Index for Nursing and Allied Health Literature via EBSCO and PsycINFO via the OVID platform.

### Eligibility Criteria

Population, Intervention, Comparison, Outcome, Study design criteria (PICOS [71]) that guided study selection and organization of data extraction for this systematic scoping review are detailed below.

#### Population

Eligible studies enrolled dyads of care recipients and support persons. The *care recipient* is a person who has an identified mental, behavioral, or physical health condition. The *support person* provides informal, unpaid care to the care recipient. Dyads are defined by existing, personal relationships like kinship or friendship, meaning that dyads of care recipients with a formal, trained health care provider (eg, their physician or an assigned trained peer mentor) were excluded. There could be more than one support person in a dyad—for example, a child with illness participating with both parents could be a *dyad*. There was no restriction on age of the dyad members, provided that the individual could consent or assent to participate.

#### Intervention

There were 3 intervention-related inclusion criteria that defined eligible dyadic eHealth interventions. First, an intervention must have intervened with both members of the dyad. Second, drawing from prevailing definitions of eHealth [72,73] and internet interventions [74], an intervention must have utilized information communication technology (including, but not limited to, the internet), with at least some intervention content delivered fully automated. Owing to this criterion, interventions that exclusively utilized technology to deliver standard clinician-provided care (eg, a therapist providing face-to-face therapy via video chat) were excluded. Third, an intervention must have provided cognitive, behavioral, educational, and/or supportive care with the primary goal to address symptom management and/or coping with the care recipients’ targeted health condition. Owing to this criterion, couples therapy that intervenes with the primary goal of improving the couple’s relationship was excluded.

#### Outcome

Records were included if they reported any objective or self-report psychosocial, health, and/or feasibility outcome for the care recipient and/or the support person. Outcomes were specified as *primary* if they were explicitly labeled as such in the record or hypotheses were explicitly specified about the outcome. Outcomes were specified as (1) an *objective* psychosocial or health measure, such as data derived via lab test (eg, hemoglobin A_1c_), diagnostic interview (eg, structured clinical interview for Diagnostic and Statistical Manual of Mental Disorders-5), or chart review (eg, documented family meeting); (2) a *self-report* psychosocial or health measure, such as data derived via self-reported questionnaire; or (3) a *feasibility/usability* measure, which may have been objective (eg, website logins) or self-reported (eg, satisfaction).

#### Study Design and Comparators

All trial designs were eligible for inclusion, including single-arm trials, feasibility trials, patient preference trials (ie, care recipient could enroll in an intervention with or without a support person), and randomized controlled trials (RCTs). There was no restriction on the type of comparison condition in RCTs. For the purposes of this review, waitlist control conditions are included under the umbrella of *usual care*. All study analytic designs were eligible for inclusion, including records that reported long-term follow-ups and secondary analyses of trial data. Records that exclusively discussed intervention development, but did not report testing of the intervention as it was intended to be used, were excluded.

### Search Methodology for Identification of Studies

The search was initially conducted by MH in January 2018 and an update was conducted in March 2019. Three broad concept categories were searched (dyads, eHealth, and psychosocial intervention), and results were combined using the appropriate Boolean operators (AND, OR). See Table 1 for search strategy. Potentially eligible records were also identified through other sources, such as via review of references of included records, communication with record authors, and a hand search of Journal of Medical Internet Research publications.

**Table 1:** Search strategy.

Concept category (combined with AND)	Search terms (combined with OR)
Dyads	dyad, dyads, dyadic, couple, couples, spouse, spouses, “Spouse”[MeSH], partner, partners, “Sexual Partners”[MeSH], parent, parents, “Parents” [MeSH], parental, guardian, guardians, “Legal Guardians”[MeSH], child, “Child”[MeSH], children, kid, kids, adolescent, adolescents, “Adolescent”[MeSH], teen, teens, teenager, teenagers family, families, “Family”[MeSH], informal caregiver, caregiver, “Caregivers”[MeSH], carer, carers
eHealth	Internet, “Internet”[MeSH], cyberspace, web, web-based, ehealth, e-health, “Telemedicine”[MeSH], mobile health, mhealth, m-health, social media, “Social Media”[MeSH], blog, blogs, mobile app, mobile application, User-Computer Interface, website, webpage
Psychosocial intervention	“Behavioral Medicine”[MeSH], psychosocial intervention, behavior therapy, “Behavior Therapy”[MeSH], cognitive therapy, “Cognitive Behavioral Therapy”[MeSH], couples therapy, “Couples Therapy”[MeSH], family therapy, “Family Therapy”[MeSH], psychoeducation, psycho-education psychoeducational, psycho-educational, “Psychology, Medical”[MeSH], “Psychology, Clinical”[MeSH]

### Study Selection

After removal of duplicate articles, study titles and abstracts were scanned by 2 of 3 coders (KS, AT, and SB) to determine whether the study met eligibility criteria. Discrepancies between coders were reviewed at a consensus meeting of all 3 coders. AT and SB retrieved full-text articles for citations that initially met the eligibility criteria. Full-text articles were read by KS, AT, or SB to make a final determination of eligibility, with ongoing coding questions reviewed at regular coder consensus meetings. Title/abstract and full-text coding were conducted using Covidence systematic review software (Veritas Health Innovation, Australia) [75]. Coders were not blind to journals or study authors during screening.

Reasons for article exclusion during the full-text review were recorded. Exclusion criteria based on PICOS criteria were (1) Not dyadic—an intervention did not intervene with a care recipient and informal support person to address the care recipient’s health condition; (2) intervention development, protocol, and/or no psychosocial, health, or feasibility outcome reported; (3) no automated components; (4) prevention study—neither dyad member had an indicated health condition at the time of enrollment (ie, there was no *care recipient*). There was 1 conference abstract that passed title/abstract screening based on review of its title, but the full abstract text was not retrievable from databases, the conference organization, or the authors. Authors indicated that abstract results were reported in a journal article of the same name, which was included in full-text coding.

### Data Extraction

Coders (AT and a trained research assistant) extracted data from the records that met eligibility criteria. Data were collected using a standardized, predefined charting form via Qualtrics. KS checked all extractions after completion by the coders. Discrepancies were resolved by tertiary review by SB, who discussed findings with KS. Articles were not blinded during data extraction. Corresponding authors for all articles were contacted and asked to review data extracted from their articles. Data were then summarized, and themes were reviewed.

## Results

### Overview

**Figure 1:**
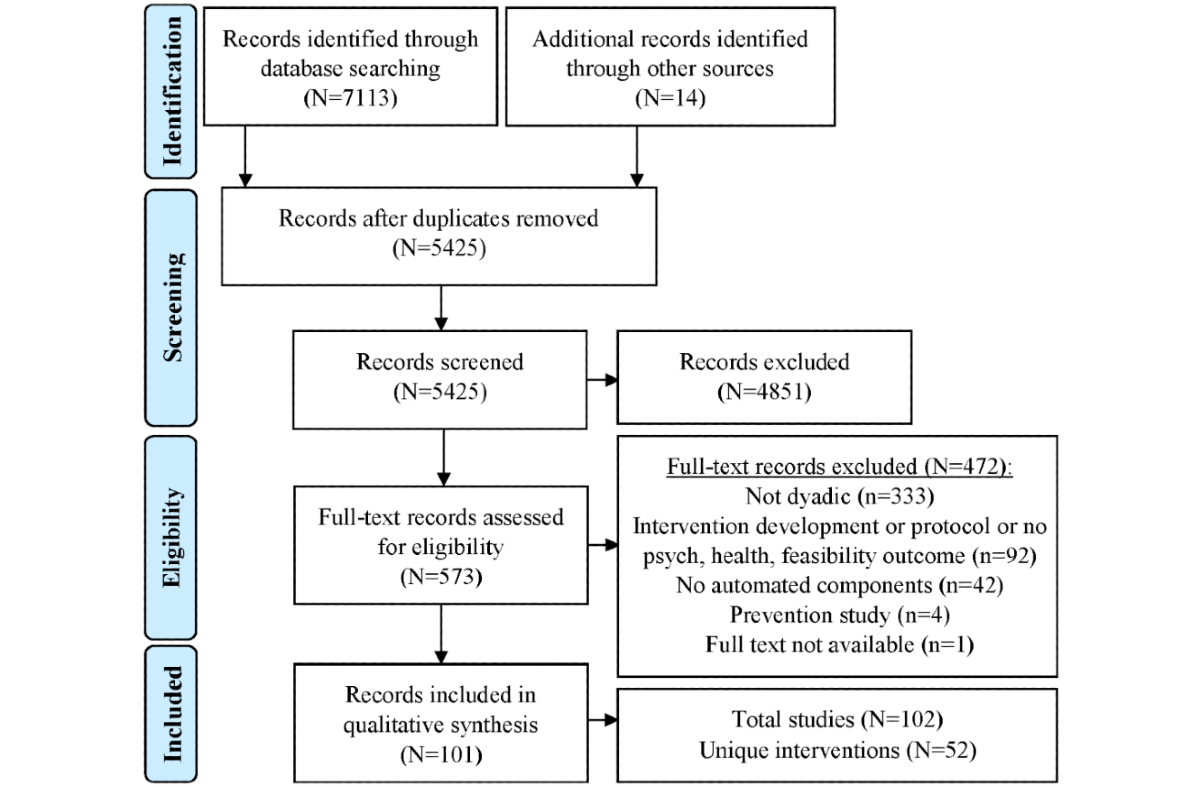
Preferred Reporting Items for Systematic Reviews and Meta-Analyses flow diagram.

The Preferred Reporting Items for Systematic Reviews and Meta-Analyses [76] flow diagram is presented in Figure 1. In total, 7113 records were identified via the search terms, and 14 were identified through other sources (including email responses from contacted authors), of which 573 were retrieved and reviewed for full-text coding. Of these, 101 records met eligibility criteria and were included in the qualitative synthesis. Data extraction is represented in Multimedia Appendix 1 [77-95,111,115-195], as well as in sortable worksheets available through the Open Science Framework by the Center for Open Science on its website [196].

Included records were published as early as 2003 [77,78]. There is a noted acceleration in the numbers of records published over the past 10 years: 22 records were published from 2003 to 2010, and 79 were published from 2011 through March 2019. Most records were full-text manuscripts (93/101, 92.1%). Across records, there were 73 unique trials—1 record [79] reported 2 separate trials. Trials were most commonly conducted in the United States (37/73, 51%), with 14 in Sweden (19%), 10 conducted in Australia (14%), and 4 in Canada (5%), among other countries of origin. In total, there were 52 unique dyadic eHealth interventions identified. The following sections present summarized findings related to study population, intervention system components, and study outcomes and design characteristics.

### Populations

A total of 72 records reported results from 52 trials of 33 unique dyadic eHealth interventions that targeted dyads in which the care recipient was a minor. For all of these trials, it was specified that a parent/guardian must be the support person. Only 1 trial [80,81] did not require that a parent/guardian participate in order for the child to participate—all other trials did require a parent/guardian to participate. These interventions most commonly targeted the care recipient’s mental health condition (10/33, 30%). Other conditions targeted included obesity (8/33, 24%), gastrointestinal disorders (4/33, 12%), pain conditions (3/33, 9%), and traumatic brain injury (2/33, 6%). In all, 3 trials targeted dyads including care recipients with 2 specific comorbidities. Conaughton et al [82] tested a transdiagnostic internet-delivered cognitive behavioral therapy for anxiety program among children with both an anxiety disorder and high functioning autism spectrum disorder; Lee et al [83] developed an mHealth program to address weight management among children with both overweight/obesity and intellectual disability; and Palermo et al [84] tested a Web-based program for children with chronic pain and sickle cell disease.

Only 1 record reported results from 1 trial of 1 intervention that specifically recruited an adolescent-young adult population (ie, care recipients ranged from mid-teens through early 30s [85]). The support person could be any close individual (eg, family member, romantic partner, or nonrelative) and was not required to enroll in order for the care recipient to participate. This study targeted care recipients who required catheterization.

A total of 28 records reported results from 21 trials of 18 interventions in which the care recipients were exclusively adults (ie, aged 18 years or older). Six of these trials (6/21, 29%) targeted recruitment to dyads of spouses/romantic partners; in the remaining 15 trials (15/21, 71%), the support person could be any close individual. Of note, the 3 trials of the *CarePartner* intervention [86-90] required that the participating support person reside outside the care recipient’s home. In all, 11 of the 21 (52%) trials of adult dyadic eHealth interventions required that both the care recipient and the support person enroll together for either to participate. Adult dyadic eHealth interventions most frequently targeted care recipients with cancer (9/18, 50%). Other conditions targeted included mental health conditions (3/18, 17%) and diabetes (2/18, 11%). Two trials targeted dyads including care recipients with 2 specific comorbidities. Both Schover et al [91] and Hummel et al [92,93] tested internet-based interventions addressing sexual dysfunction secondary to cancer.

### Intervention Information

#### Intervention System Components

Interventions tended to deliver content, intervene, and engage users through multiple components: the median number of components was 3 (*M*=3.03), ranging from 1 to 6 components. The 3 most commonly utilized components were browser-based components, human telephone contact, and asynchronous communication portals. Browser-based components, or webpages, were used in 44 of 52 (85%) interventions. Human telephone contact, most commonly contact with a therapist or research staff person, was used in 25 interventions (25/52, 48%). Asynchronous communication portals, most commonly used to contact a therapist, research staff person, or other intervention participants via a communication portal embedded within the program such as encrypted email portal or a discussion board, were used in 22 interventions (22/52, 42%).

A total of 40 interventions (40/52, 77%) required human support as they included a component that required therapist or research staff effort (ie, videoconferencing, human telephone contact, human emails/SMS, asynchronous communication portal, synchronous chat room, or in-person sessions).

#### Dyad Participation and Content Target

In all, 21 interventions were intended to be used entirely separately by care recipients and support persons (21/52, 40%). Interventions were almost as commonly intended to be used with care recipients and support persons accessing some aspects of the intervention together and other aspects separately (20/52, 38%). Fewer interventions were intended to be used by the dyad entirely together (6/52, 12%). In some cases (5/52, 10%), it was not specified how an intervention was intended for use by care recipients and support persons—either because it was unspecified in the record or because there was no specific way that dyads were expected to interact with the intervention.

Most interventions (38/52, 73%) had content that was exclusively intended to be accessed by support persons, and most had content that was exclusively intended to be accessed by care recipients (38/52, 73%). A total of 32 interventions (32/52, 62%) had shared content that was accessible to both the care recipients and support persons. In all, 14 interventions (14/52, 27%) included all 3 types of content (ie, for care recipients only, for support persons only, and shared).

### Outcomes, Study Design, and Comparators

Most studies (63/102, 61.8%) specified one or more primary outcomes for care recipients and 22 of 102 (21.6%) specified one or more primary outcomes for support persons. In all, 6 studies (6/102, 5.9%) reported no outcomes for care recipients and 35 (35/102, 34.3%) reported no outcomes for support persons. Among the 63 studies specifying primary outcomes for care recipients, 23 (37%) included objective primary psychosocial or health outcomes, 43 (68%) included self-report primary psychosocial or health outcomes, and 3 (5%) included feasibility/usability primary outcomes (see Methods, Eligibility Criteria, Outcome section for definitions). Among the 33 studies specifying other outcomes for care recipients, 7 (21%) included objective outcomes, 14 (42%) included self-report outcomes, and 26 (79%) included feasibility/usability outcomes. Among the 22 studies specifying primary outcomes for support persons, 3 (14%) included objective primary outcomes, 18 (82%) included self-report primary outcomes, and 2 (9%) included feasibility/usability primary outcomes. Among the 46 studies specifying other outcomes for support persons, 1 (2%) included objective outcomes, 21 (46%) included self-report outcomes, and 34 (74%) included feasibility/usability outcomes.

Trials of dyadic eHealth interventions were most commonly RCTs (44/73, 60%). Among the 44 RCTs, most (22/44, 50%) compared the dyadic eHealth intervention with usual care alone; 8 (18%) with an educational website or internet-delivered resources; 4 (9%) with an in-person version of the intervention; 2 (5%) with an intervention delivered exclusively to the care recipients (ie, no support person intervention); 2 (5%) with an intervention delivered exclusively to support persons (ie, to parents alone); and 6 (14%) with another kind of intervention. Less common study designs were single-arm trials (23/73, 32%), patient preference trials (3/73, 4%), parallel-group RCTs (2/73, 3%), or observational studies (1/73, 1%).

Among studies from RCTs that specified one or more primary outcomes for care recipients, 95% (19/20) of dyadic eHealth interventions demonstrated at least one or more statistically superior outcomes compared with usual care and 20% (1/5) with an educational website or internet-delivered resources. Positive findings for primary outcomes were found in a trial comparing a combination of 2 interventions with either of the singular interventions. In all, 2 RCTs compared a dyadic eHealth intervention with a parent-only eHealth intervention: 1 trial demonstrated better outcomes for care recipients in the dyadic condition [94] and 1 trial demonstrated better outcomes for care recipients in the parent-only condition [95]. There were no significant differences for any care recipient primary outcomes when dyadic eHealth interventions were compared with an in-person version of the intervention (n=3 trials), an intervention delivered exclusively to the care recipients (n=1), or another kind of intervention (n=2).

Among RCTs that specified one or more primary outcomes for support persons, 60% (3/5) of dyadic eHealth interventions demonstrated at least one or more statistically superior outcomes compared with usual care and 67% (2/3) with an educational website or internet-delivered resources. In 1 trial, parents’ involvement in their child’s care was superior among parents in a parent-only intervention relative to the dyadic intervention [95]. There were no significant differences for any support person primary outcomes when dyadic eHealth interventions were compared with an in-person version of the intervention (n=2 trials) or another kind of intervention (n=1).

Among single-arm trials that compared preintervention to postintervention levels of one or more specified primary outcomes, 93% (13/14) showed at least one significantly improved outcome for care recipients and 100% (3/3) for support persons. The 1 patient preference trial that specified primary outcomes demonstrated better outcomes for care recipients who elected to enroll with a support person compared with those who enrolled by themselves [88].

## Discussion

### Principal Findings

This review provides the first comprehensive summary of dyadic interventions utilizing eHealth approaches to provide psychosocial care to care recipients and their support persons together as a unit. Three research questions guided the review: what populations had been targeted by these interventions, how technologies have been utilized in these interventions, and approaches these interventions have taken to intervene with the dyad. Of over 7000 reviewed records, 101 met eligibility criteria and described 52 unique dyadic eHealth interventions. In all, 33 of the unique interventions were conducted among dyads of children and their supporting parent, and 18 of the interventions were conducted among adult dyads with diverse relationships. Less than one-fourth of all interventions were fully automated. When a primary outcome was specified, the results of the dyadic eHealth intervention tended to be positive for both care recipients and support persons, although less than one-fourth of studies specified a primary outcome for support persons. One-third reported no outcomes for support persons at all.

This review reveals that there is substantial, diverse, and growing literature supporting dyadic eHealth interventions. This robust literature demonstrates a broad range of strategies for delivering interventions to dyads via eHealth and also identifies several significant gaps in the science. By summarizing broadly across the literature, findings highlight areas of research opportunities for developing dyadic eHealth interventions for novel populations and increasing access to dyadic care. Findings also demonstrate broad variability in approaches of intervening with dyads, with open questions remaining regarding the necessary and sufficient components that define a *dyadic intervention*.

### Populations Targeted by Dyadic Electronic Health Interventions

The literature base for pediatric dyadic eHealth interventions was relatively more established compared with the more nascent rise in dyadic eHealth interventions for adult dyads. There were almost twice as many unique eHealth interventions and trials for parent-child dyads compared with those for dyads with adult care recipients. Only 2 of 18 adult dyadic eHealth interventions had been tested in more than 1 trial. This is compared with 7 of 33 pediatric dyadic eHealth interventions that were tested in more than 1 trial, and 3 interventions were tested in 4 or more trials. This discrepancy may be due in part to the fact that pediatric dyadic eHealth interventions started to be published much earlier than adult interventions: about one-fourth of the pediatric records were published before 2010, whereas only 2 records on an adult dyadic eHealth intervention were published before 2010. This finding also reflects the more long-standing emphasis on family-centered care in pediatric health care [96-98] and the increasing acceptance of family-centered care among adult health care delivery [99-101].

There was also a discrepancy between pediatric and adult dyadic eHealth interventions in terms of the care recipient conditions targeted by the interventions. Although over half of adult dyadic eHealth interventions addressed coping with the care recipients’ cancer treatment and side effects, there were no pediatric interventions specifically addressing cancer. In contrast, pediatric interventions commonly targeted obesity and pain, whereas no adult interventions targeted these specific conditions. Cancer, obesity, and chronic pain affect individuals of all ages and their families and are strongly influenced by heritable and shared lifestyle factors within families. As such, it is worth considering how dyadic eHealth interventions might be extended to address pediatric and adolescent/young adult cancer survivorship, as well as obesity and chronic pain among adults.

One population notably missing from representation is older adult dyads (eg, care recipients and/or support persons over age 65 years). In the context of our eligibility criteria, no interventions were specifically tailored to older adult needs, no trials recruited an exclusively older adult population, and no records specifically reported intervention effects among older participants. Older adult care recipients are most likely to be cared for by their spouses who frequently have health limitations of their own [7], meaning that dyadic care is particularly important for older adults. Older support persons are as interested in eHealth resources as younger support persons [102]. In actual practice, however, older support persons are less likely to access internet-based caregiving resources than younger ones [103,104]. This discrepancy may in part be because of older adults having difficulty navigating eHealth resources [105], which emphasizes the importance of developing dyadic eHealth tools specifically with older users in mind.

### Use of Electronic Health Technologies

Technology that automates care is integral to extend the reach of dyadic interventions by overcoming current logistical and financial barriers [6,38,106]. More than three-fourths of dyadic eHealth interventions identified in this review, however, utilized human support. In addition, 2 of the 3 most commonly used intervention system components require human support (ie, by telephone or asynchronous communication portal). Human involvement was not always extensive. For instance, with Web-based Management of Adolescent Pain, Palermo et al [84] report expressly instructing therapists to spend no more than 5 min to respond to a participant message. Importantly, such minimal human support may be sufficient to enhance clinical outcomes: in one of their studies, Anderson et al [79] demonstrated that dyads of anxious youths and their parents reported comparable working alliance with their therapist in a minimal-touch Web-based version of an intervention as dyads receiving the intervention in traditional face-to-face care.

There may be important rationale to include human support in a dyadic eHealth intervention, such as to increase perceived acceptability, user engagement, and clinical outcomes [107-109]. Progressing capabilities of technology to provide automated and personalized feedback, however, suggest that discrepancies in implementation and clinical outcomes will continuously narrow between human-supported and automated interventions [38,110]. Indeed, it appears that dyads may *vote with their feet* toward increasing automation—Schover et al [91] reported that their recruitment rate tripled when dyads were guaranteed access to a Web-based intervention as opposed to being randomized between the Web-based intervention or to the same intervention content delivered by telephone with a nurse. In their pilot of a Web-delivered adaptation of a previously nurse-delivered coping program, Northouse et al [111] found that enrollment rates for the Web version were lower than enrollment rates into prior trials for the nurse-delivered version; however, the retention rate for the Web version was higher than retention rate for the nurse-delivered version. As technological capacity—and society’s expectations for it—increase, the relative advantages of clinician support compared with highly responsive technologies should be carefully considered to ensure greatest efficacy and reach of dyadic interventions.

### Approaches to Intervening With the Dyad

In this review, we utilized a broad definition to identify dyadic interventions—namely, an intervention that intervened with both a care recipient and (at least one) informal support person to provide comprehensive treatment for the care recipient’s health condition. About 1 in 4 identified dyadic eHealth interventions appeared to address the unique needs of each individual in the dyad, while also addressing relational factors between the dyad. These interventions both (1) included content that was uniquely tailored for and exclusively accessible by care recipients and support persons separately, in addition to shared content available to both users; and (2) were either intended to be utilized both together and separately or left up to the dyads how to interact with the intervention. Addressing relational factors between dyad members is likely a key element to delivering synergistic benefits beyond those from individual interventions, as they enhance natural support between dyad members [112-114]. Previous literature suggests that dyadic interventions addressing these relational aspects of coping with illness are more effective than primarily informational interventions [4].

An example of a dyadic eHealth intervention that addresses both individual needs and the dyadic relationship is the Schizophrenia Online Access to Resources intervention [115-117]. This intervention addresses unique concerns of both dyad members—for example, with unique therapy forums for the individuals with schizophrenia only and support persons only, as well as addressing shared skills for the whole family—for example, shared content regarding developing a supportive safety net together. Across their manuscripts, Rotondi et al [115-117] establish rationales for the use of dyadic and eHealth approaches: a dyadic approach was utilized because family-based therapy is the gold standard for schizophrenia, given the detrimental effects of a stressful family environment on worsening patients’ symptom severity. An eHealth approach was utilized given that traditional in-person family-based schizophrenia interventions have had low uptake and retention, and authors reasoned that eHealth programs would be more accessible to families affected by schizophrenia.

### Limitations and Future Directions

There are limitations to both the body of literature summarized and the methods we used to summarize this literature. Regarding the former, less than two-thirds of the 52 unique dyadic eHealth interventions have been tested for efficacy in an RCT. Among RCTs, over half compared against usual care or waitlist control alone. Although the usual care comparator may be an important first step toward demonstrating clinical benefits of an intervention, these trials leave important questions about the unique benefits of dyadic interventions unanswered. For example, trials are needed that compare outcomes for both patients and support persons when provided a dyadic eHealth intervention versus individual eHealth interventions for patients and for support persons alone. Such trials would begin to demonstrate whether, and under what circumstances, dyadic eHealth interventions are superior to individually delivered interventions. eHealth interventions are better suited for such trials than face-to-face interventions, given large-scale recruitment is more feasible. In addition, participants can be randomized to receive only part of a larger intervention: for instance, 1 dyad receives the full dyadic eHealth intervention, whereas in another dyad, only the support person receives applicable content from the full intervention. These data will be necessary to justify the added costs and complication of intervening with both care recipients and support persons together.

Another substantial limitation of this literature is the fact that one-third of all studies reported no outcomes for support persons. This issue was identified in one of the earliest reviews of dyadic psychosocial interventions published almost 15 years ago [1]. It is possible that support person outcome data were collected but not reported in these records. Regardless, omitting support person data is a significant missed opportunity to demonstrate the effects of an intervention to roughly half of its users. Given the extensive care responsibilities support persons already assume and the psychosocial and physiological strain of those responsibilities, intervention developers should carefully consider whether the benefits of including a support person in an intervention ultimately outweigh the costs to that support person. Reporting outcomes for support persons are critical to demonstrate that these interventions, at minimum, do no harm to them.

Although the breadth of this review is a strength, the lack of common vocabulary and established criteria that define dyadic interventions made this review challenging to complete. Pediatric interventions, in particular, may be more commonly described as *family based* rather than *dyadic*. To best ensure we captured all interventions that met our broad definition of dyadic eHealth interventions, we carefully developed a search strategy with our medical librarian. It remains possible, however, that we missed interventions that meet our core definitional qualities of dyadic eHealth interventions but use a different vocabulary. Ultimately, to advance the science on dyadic interventions, reaching consensus on ideal qualities of dyadic interventions will be helpful to guide development, assessment, and dissemination of this model of care.

In addition, as a scoping review covering broad literature, we were limited to capturing only superficial components of these interventions. In particular, we were unable to more extensively describe the nature of content delivered by the interventions, which would afford a more nuanced understanding of how interventions address dyad members’ unique needs and their relational needs. Given the breadth of data extracted from the volume of records, we also were unable to present detailed results related to study outcomes and designs across subsets of populations, health conditions, or strategies. Finally, we did not extract sample sizes from the studies or location where the intervention was received by participants (eg, home and health care facility). Ideally, this review highlights the breadth of the dyadic eHealth literature and the opportunities for more specific systematic reviews on pertinent subtopics.

### Conclusions

This first comprehensive review of dyadic eHealth interventions identified the substantial and rapidly growing literature describing the use of these interventions across a broad range of populations. Collating this robust literature will hopefully serve as a resource for intervention developers to identify models most likely to be effective given the goals and requirements of a particular intervention, rather than models that have been typically used within a particular population. Although the literature on dyadic eHealth interventions is robust, there are significant gaps. For instance, few studies reported outcomes for support persons, data which are essential to justifying their inclusion in interventions. Other gaps highlight important future research needs: for instance, development of interventions designed for older adults and trials comparing the relative efficacy of dyadic and individual eHealth interventions. As technology advances, further personalizing and automating dyadic eHealth interventions will help to increase their scalability, and ultimately, their likelihood of benefitting all who are affected by illness.

## Appendix

Multimedia Appendix 1 Study Information.

## Abbreviations

eHealth: electronic health
PICOS: Population, Intervention, Comparison, Outcome, Study
RCT: randomized controlled trial
