# Supplementary material for: Dyadic Psychosocial eHealth Interventions: Systematic Scoping Review
Source: J Med Internet Res. 2020 Mar 4;22(3):e15509. doi: 10.2196/15509 (PMC7081137; doi:10.2196/15509)
Supplement: Multimedia Appendix 1 [file jmir_v22i3e15509_app1.pdf]

Appendix 1. Study Information

Description			Population		Intervention			Outcomes		Study design & comparator	
Intervention Name	Study	Country	CR Age Group / Condition	SP Relationship / Required	System components	Dyad participation	Content target	CR Outcomes	SP Outcomes	Trial design	Comparator
Health Information technology (HIT) enhanced self-monitoring of blood glucose + Family-centered goal planning	Hannon 2018 [118]	USA	Pediatric / Diabetes	Parent / Y	1,2,8, 10,13	Separate and together	Shared CR only	**HbA1c**[+HIT alone, +Family alone]	None	RCT	HIT enhanced self-monitoring of blood glucose alone; Family-centered goal planning alone
Steps to Active Kids with Diabetes (STAK-D)	Quirk 2018 [119]	UK	Pediatric / Diabetes	Parent / Y	1,4,12, 13	Entirely separate	CR only SP only	Feasibility and acceptability (rate of recruitment, adherence, retention, implementation fidelity, adverse events, and data completion); Self-reported physical activity; Objective physical activity; Self-efficacy for physical activity	None	RCT	UC
The 3Ms (Medicine, Meter, Meals)	Ellis 2017 [95]	USA	Pediatric / Diabetes	Parent / Y	1	Entirely separate	CR only SP only	**Glycemic control**[-Parent-only]	**Knowledge**[=Parent-only, +Attn]**, motivation**[=Both]**, self-efficacy**[=Both]**, and direct parental monitoring**[-Parent-only, =Attn]	RCT	Parent-only intervention; Attention control
Coping skills intervention	McCormick 2010 [120]	USA	Pediatric / Gastrointestinal disorder	Parent / Y	1,7,11, 13	Separate and together	Shared CR only SP only	**Physical abdominal pain symptoms**[=]**; Other somatic symptoms**[=]	None	RCT	UC
	Reed-Knight 2012 [121]							Web participation rates; Collection of post-treatment data	Web participation rates; Collection of post-treatment data		

Description			Population		Intervention			Outcomes		Study design & comparator	
Intervention Name	Study	Country	CR Age Group / Condition	SP Relationship / Required	System components	Dyad participation	Content target	CR Outcomes	SP Outcomes	Trial design	Comparator
Exposure-based Internet CBT	Bonnert 2014 [122]	Sweden	Pediatric / Gastrointestinal disorder	Parent / Y	1,7,8,10	Entirely separate	Shared CR only SP only	**Global gastrointestinal symptoms^{+}**	None	Single-arm	N/A
	Bonnert 2017 [123]	Sweden	Pediatric / Gastrointestinal disorder	Parent / Y	1,7,8,10	Entirely separate	Shared CR only SP only	**Global gastrointestinal symptoms^{+}**	None	RCT	UC
	Bonnert 2018 [124]							**Secondary analysis examining avoidance behavior^{+} and stress$^{=}$ as mediators for gastrointestinal symptom improvement**	None		
	Sampaio 2019 [125]							**Quality-adjusted life years^{+}**	None		
	Bonnert 2019 [126]	Sweden	Pediatric / Gastrointestinal disorder	Parent / Y	1,8,9,10	Entirely separate	Shared CR only SP only	**Pain intensity^{+}**	Monitoring behavior, protective behavior, minimizing behavior, and distraction from pain in relation to child's symptoms	Single-arm	N/A
Internet-delivered CBT	Lalouni 2017 [127]	Sweden	Pediatric / Gastrointestinal disorder	Parent / Y	1,10	Separate and together	Shared SP only	**Global gastrointestinal symptoms^{+}**	Work absence as a result of child's abdominal problems; Adult responses to children's symptoms; Depressive symptoms; Anxiety symptoms	Single-arm	N/A
	Lalouni 2018 [128]	Sweden	Pediatric / Gastrointestinal disorder	Parent / Y	1,7,8,10	Entirely separate	CR only SP only	**Gastrointestinal symptom severity^{+}**	Responses to child's pain behavior (protect, monitor)	RCT	UC
U-CAN-POOP-TOO	Ritterband 2003 [77]	USA	Pediatric / Gastrointestinal disorder	Parent / Y	1,12	Entirely together	Shared	**# accidents per week^{+}; # bowel movements in toilet per week^{+}; Bathroom use without parental prompts^{+}; Bathroom use with parental prompts$^{=}$**	Knowledge about encopresis; Experience using the website; Website usage data	RCT	UC
	Ritterband 2008 [129]	USA	Pediatric / Gastrointestinal disorder	Parent / Y	1	Entirely together	Shared	Utility; Perceived impact; Adherence; # fecal accidents; # bowel movements in toilet; Pain during defecation; Website usage data	Utility; Perceived impact; Adherence; Website usage data	Observational	N/A
	Ritterband 2013 [130]	USA	Pediatric / Gastrointestinal disorder	Parent / Y	1	Entirely together	Shared	**# fecal accidents^{+}; Success rates (based on fecal accidents)$^{=}$; Cure rates (based on fecal accidents)$^{=}$**	Knowledge about encopresis; Perceived usability, likability, usefulness, understandability, and convenience; Website usage data	RCT	UC

Description			Population		Intervention			Outcomes		Study design & comparator	
Intervention Name	Study	Country	CR Age Group / Condition	SP Relationship / Required	System components	Dyad participation	Content target	CR Outcomes	SP Outcomes	Trial design	Comparator
BIP Anxiety	Jolstedt 2018 [94]	Sweden	Pediatric / Mental health condition	Parent / Y	1,10	Separate and together	Shared SP only	**Anxiety symptom severity**$^{+}$	Tx expectancy/credibility; Adherence; Engagement; Satisfaction; Absence from work; Productivity losses at work	RCT	Internet-delivered Child-Directed Play (Parent only)
BIP OCD	Lenhard 2014 [131]	Sweden	Pediatric / Mental health condition	Parent / Y	1,7,10	Separate and together	CR only SP only	**OCD symptom severity**$^{+}$	Adherence	Single-arm	N/A
	Lenhard 2016 [132]							Qualitative feedback on: General positive and negative experiences; Experiences of the treatment (Tx) process; Experiences of the usefulness of the Tx; Unmet expectations and suggestions for improvement	None		
	Lenhard 2017a [133]	Sweden	Pediatric / Mental health condition	Parent / Y	1,2,7,10	Separate and together	CR only SP only	**Treatment responder rate**$^{+}$	None	RCT	UC
	Lenhard 2017b [134]							**OCD symptom severity**$^{+}$	Parental accommodation of OCD behaviors		
	Aspvall 2018 (BIP OCD Junior) [135]	Sweden	Pediatric / Mental health condition	Parent / Y	1,7,10	Separate and together	CR only SP only	**OCD symptom severity**$^{+}$	Tx credibility; Satisfaction	Single-arm	N/A
BIP SOFT	Nordh 2017 [136]	Sweden	Pediatric / Mental health condition	Parent / Y	1,7,9, 10,13	Separate and together	CR only SP only	**Global symptom severity**$^{+}$	Productivity costs associated with child's illness; Usefulness, acceptability, and satisfaction of Tx	Single-arm	N/A
BIP TIC ERP / BIP TIC HRT	Andrén 2019 [137]	Sweden	Pediatric / Mental health condition	Parent / Y	1,7,8,9, 10	Entirely separate	CR only SP only	**Global tic severity**$^{+}$ **/ Global tic severity**$^{=}$	Treatment credibility; Satisfaction	Parallel-group RCT – within-group pre-post comparisons	N/A

Description			Population		Intervention			Outcomes		Study design & comparator	
Intervention Name	Study	Country	CR Age Group / Condition	SP Relationship / Required	System components	Dyad participation	Content target	CR Outcomes	SP Outcomes	Trial design	Comparator
BRAVE-ONLINE	Spence 2006 [138]	Australia	Pediatric / Mental health condition	Parent / Y	1,9,13	Entirely separate	CR only SP only	Anxiety diagnoses; Clinician severity ratings and questionnaire measures; Clinical significance of symptom changes	None	RCT	UC and fully in-person CBT
	March 2009 [139]	Australia	Pediatric / Mental health condition	Parent / Y	1,7,8,9, 12	Separate and together	CR only SP only	Tx expectancy and credibility; # diagnoses; Clinician severity rating; Anxiety symptoms	None	RCT	UC
	Spence 2011 [140]	Australia	Pediatric / Mental health condition	Parent / Y	1,7,8,9	Entirely separate	CR only SP only	**# diagnoses$^{+UC, =in\text{-}person}$; Clinician severity rating$^{+UC, = in\text{-}person}$; Global functioning$^{+UC, = in\text{-}person}$**	None	RCT	UC and in-person BRAVE
	Anderson 2012 (study 1) [79]	Australia	Pediatric / Mental health condition	Parent / Y	1,7,8,9	Entirely separate	CR only SP only	**Working alliance$^{=}$**	**Working alliance$^{=}$**	RCT	Clinic-based Tx
	Anderson 2012 (study 2) [79]	Australia	Pediatric / Mental health condition	Parent / Y	1,7,8,9	Entirely separate	CR only SP only	**Global functioning^{+}**	None	Single-arm	N/A
	Conaughton 2017 [82]	Australia	Pediatric / Mental health condition & Other (Autism Spectrum Disorder)	Parent / Y	1,7,8,9	Entirely separate	CR only SP only	# diagnoses; Clinical severity rating; Global functioning; Anxiety symptoms; Internalizing behaviors	None	RCT	UC
	Stasiak 2018 [141]	New Zealand	Pediatric / Mental health condition	Parent / Y	1,7,8,9, 12	Separate and together	CR only SP only	**# anxiety disorders^{+}; severity of anxiety disorders^{+}**	Program satisfaction and completion	Single-arm	N/A
CATCH-IT 3	Van Voorhees 2015 [80]	USA	Pediatric / Mental health condition	Parent / N	1,7,13	Entirely separate	CR only SP only	Adherence (reported from initial trial data; full trial to report depressive episodes, depressed mood, functional status)	Adherence (reported from initial trial data; full trial to report depressed mood)	RCT	General health education
	Gladstone 2018 [81]							**Time to event for depressive episode$^{=}$; Depressive symptoms$^{=}$**	None		
Internet cognitive-behavioral skills-based program	Keller 2009** [142]	USA	Pediatric / Mental health condition	Parent / Y	1,7	Separate and together	Shared SP only	**Anxiety symptoms^{+}**	Mother's anxiety; Emotional experiences	RCT	UC

Description			Population		Intervention			Outcomes		Study design & comparator	
Intervention Name	Study	Country	CR Age Group / Condition	SP Relationship / Required	System components	Dyad participation	Content target	CR Outcomes	SP Outcomes	Trial design	Comparator
Internet-delivered cognitive behavioral therapy for children with anxiety disorders	Vigerland 2016 [143]	Sweden	Pediatric / Mental health condition	Parent / Y	1,7,10	Separate and together	Shared SP only	**Clinician severity rating^{+}**	Tx satisfaction	RCT	UC
OCD? Not me!	Rees 2016 [144]	Australia	Pediatric / Mental health condition	Parent / Y	1,8	Entirely separate	CR only SP only	**# OCD symptoms^{+}; OCD severity^{+}**	None	Single-arm	N/A
Parent-Adolescent Conflict Training (PACT)	Carpenter 2004 [145]	USA	Pediatric / Mental health condition	Parent / Y	1	Entirely separate	CR only SP only	Compliance; Daily log frequency; Participation rate across time; Satisfaction	Compliance; Daily log frequency; Participation rate across time; Satisfaction	Single-arm	N/A
Families Improving Together (FIT)	Wilson 2019 [146]	USA	Pediatric / Obesity	Parent / Y	1,7,8,9, 13	Separate and together	Shared SP only	None	**Relation between online engagement (login rates, length of sessions) with family retention in study**	RCT	Comprehensive health education program
Fun, Food, and Fitness Project	Baranowski 2003 [78]	USA	Pediatric / Obesity	Parent / Y	1,4,7,9, 13	Separate and together	Shared CR only SP only	BMI; Waist circumference; Physical activity; Total calorie intake; % calories from fat; Fruit and vegetable consumption; Sweetened beverage consumption; Water servings; Physical activity preferences; Sweetened beverage preferences	None	RCT	Day camp and educational control website
Health Hawks + FITNET / Health Hawks + FITNET and Skype	Tripicchio 2017 [147]	USA	Pediatric / Obesity	Parent / Y	2,4,13 (5 in Skype version only)	Separate and together	Shared CR only SP only	**BMI$^{=}$ / BMI^{+}**	Feasibility, Engagement, Retention	Parallel-group RCT – within-group pre-post comparisons	N/A
Mobile Health Information System (HIS)	Büchter 2014* [148]	Switzerland	Pediatric / Obesity	Parent / Y	2,4	Separate and together	Shared CR only	Usefulness	Usefulness	Single-arm	N/A
	Kowatsch 2014 [149]							Perceived usefulness, ease of use, enjoyment of use	Perceived usefulness, ease of use, enjoyment of use		
	Xu 2014 [150]							Duration of application use, perceived usefulness of application features	Duration of application use, perceived usefulness of application features		

Description			Population		Intervention			Outcomes		Study design & comparator	
Intervention Name	Study	Country	CR Age Group / Condition	SP Relationship / Required	System components	Dyad participation	Content target	CR Outcomes	SP Outcomes	Trial design	Comparator
Health Improvement Program for Teens (HIP-Teens)	White 2004 [151]	USA	Pediatric / Obesity	Parent / Y	1,9,10, 13	Separate and together	Shared CR only SP only	**Mediation analyses for: % body fat; Body weight; BMI**	**Mediational analyses for: % body fat; Body weight; BMI**	RCT	Educational Internet resources
	Williamson 2005 [152]							**Preliminary results: % body fat^{+}; Body weight$^{=}$; BMI^{+}**	**Preliminary results: % body fat$^{=}$; Body weight^{+}; BMI^{+}**		
	Williamson 2006 [153]							**Long-term follow-up: % body fat^{+}; Body weight$^{=}$; BMI^{+}**	**Long-term follow-up: % body fat$^{=}$; Body weight^{+}; BMI$^{=}$**		
School-based Weight Management Program	Lee 2017 [83]	Hong Kong	Pediatric / Obesity & Other (mild intellectual disability)	Parent / Y	1,2,7,9, 10,13	Separate and together	Shared CR only SP only	**Body weight$^{=}$; BMI$^{=}$; Skinfold thickness$^{=}$; Food pyramid knowledge$^{=}$; Sports pyramid knowledge^{+}; Snack choice tests^{+}**	Preferred cooking method	RCT	UC
Web-based application of family-based behavioral treatment	Moore 2011** [154]	USA	Pediatric / Obesity	Parent / Y	1	Entirely together	Shared	BMI; Global health status; Diet (fruit and vegetable consumption, sweets consumption); Physical activity	Knowledge of behavioral weight control skills; Treatment satisfaction	RCT	UC
MySteps	Mâsse 2015 [155]	Canada	Pediatric / Obesity	Parent / Y	1,4,7,8, 9	Entirely separate	CR only SP only	**Relation between adherence and individual factors (Theory of Planned Behavior constructs, Self-Determination Theory constructs) and household factors (family practices, parenting practices, family environment, food/soft drink availability)**	None	Single-arm	N/A
	Tu 2017 [156]							**Relation between adherence (participation and self-monitoring) and BMI; Waist circumference$^{=}$**	None		
DARWeb	Nieto 2015 [157]	Spain	Pediatric / Pain	Parent / Y	1,7,8,9	Entirely separate	CR only SP only	Completion rate; Time to completion; Satisfaction with intervention elements and intervention as a whole; Ideas for improving the intervention; Burden to complete the intervention; Changes to pain perception	Completion rate; Time to completion; satisfaction with intervention elements and intervention as a whole; Ideas for improving the intervention; Burden to complete the intervention	Single-arm	N/A

Description			Population		Intervention			Outcomes		Study design & comparator	
Intervention Name	Study	Country	CR Age Group / Condition	SP Relationship / Required	System components	Dyad participation	Content target	CR Outcomes	SP Outcomes	Trial design	Comparator
Move it Now	Voerman 2015** [158]	Netherlands	Pediatric / Pain	Parent / Y	1,7,9	Entirely separate	CR only SP only	**Pain intensity at this moment^{+}; Pain intensity of the worst pain today^{+}**	None	RCT	UC
WebMAP	Palermo 2009 [159]	USA	Pediatric / Pain	Parent / Y	1,10	Entirely separate	CR only SP only	**Activity limitations^{+}; Pain intensity^{+}**	Parental response to pain behavior (parental protectiveness); Treatment acceptability and satisfaction	RCT	UC
	Long 2009 [160]							Module and program ratings; Perceived usefulness; Appearance; Ease of use; Program use (interactivity, engagement)	Module and program ratings; Perceived usefulness; Appearance; Ease of use; Program use (interactivity, engagement)		
	Fales 2015 [161]							**Total sleep time$^{=}$; Sleep efficiency$^{=}$; Subjective sleep quality$^{=}$**	None		
	Law 2015 [162]	USA	Pediatric / Pain	Parent / Y	1,10	Entirely separate	CR only SP only	**Headache frequency$^{=}$**	Parental response to pain behavior (parental protectiveness)	RCT	Specialty headache Tx
	Palermo 2015 [163]	USA (also recruited in Canada)	Pediatric / Pain	Parent / Y	1,10	Entirely separate	CR only SP only	**Functional disability^{+}; Pain intensity^{+}**	None	RCT	Internet-delivered education
	Palermo 2016 [164]							**Daily activity limitations$^{=}$**	Impact of caring for a child with pain; Depressive symptoms; Anxiety; Pain catastrophizing; Self-blame; Partner relationship; Social functioning; Parental role strain		
	Fisher 2017 [165]							**Secondary analysis comparing dyads in agreement on treatment goals vs. not: Pain intensity$^{=}$; Pain-related disability$^{=}$**	None		
	Law 2017 [166]							**Secondary analysis using latent growth modeling: Child disability$^{+\dagger}$**	**Parent distress$^{+\dagger}$; Maladaptive parenting behavior^{+}**		
	Alberts 2018 [167]							**Secondary analysis examining relation of treatment outcomes (activity limitations, pain intensity) with adolescent treatment engagement**	**Secondary analysis examining relation of treatment outcomes (activity limitations, pain intensity) with parent treatment engagement**		
	Palermo 2018 [84]	USA	Pediatric / Pain & Other (sickle cell disease)	Parent / Y	1,10	Entirely separate	CR only SP only	Rates of recruitment retention, outcome measure completion; Engagement and adherence; Acceptability	Rates of recruitment, retention, outcome measure completion; Engagement and adherence; Acceptability	RCT	Internet-delivered education

Description			Population		Intervention			Outcomes		Study design & comparator	
Intervention Name	Study	Country	CR Age Group / Condition	SP Relationship / Required	System components	Dyad participation	Content target	CR Outcomes	SP Outcomes	Trial design	Comparator
CAPS (Counselor-Assisted Problem-Solving)	Narad 2015 [168]	USA	Pediatric / Traumatic brain injury	Parent / Y	1,5,13	Not specified	Shared	**Parent-teen conflict$^{=}$; Effective family communication$^{=}$; Family problem solving$^{=}$**	**Parent-teen conflict$^{=}$; Effective family communication$^{=}$; Family problem solving$^{=}$**	RCT	Internet resource comparison
Online Family Problem Solving (FPS) / Teen Online Problem Solving (TOPS)	Wade 2005 [169]	USA	Pediatric / Traumatic brain injury	Parent / Y	1,5,13	Entirely together	Shared	**Child behavior problems^{+}; Depressive symptoms$^{=}$; Metacognitive skills$^{=}$**	**Injury-related stress and burden^{+}; Parenting stress^{+}; Depression^{+}; Distress^{+}; Anxiety$^{=}$**	Single-arm	N/A
	Wade 2006a [170]	USA	Pediatric / Traumatic brain injury	Parent / Y	1,5,13	Entirely together	Shared	None	**Problem-solving skills$^{=}$; Distress^{+}; Depression^{+}; Anxiety^{+}**	RCT	Internet resource condition
	Wade 2006b [171]							Child behavior problems; Social competence; Website evaluation	None		
	Wade 2008 [172]	USA	Pediatric / Traumatic brain injury	Parent / Y	1,5,13	Entirely together	Shared	**Behavioral problems$^{=}$; Executive functioning$^{=}$**	**Distress$^{=}$; Depression^{+}**	Single-arm	N/A
	Wade 2009 [173]							Website evaluation; Satisfaction	Website evaluation; Satisfaction		
	Wade 2011 [174]	USA	Pediatric / Traumatic brain injury	Parent / Y	1,5,13	Entirely together	Shared	**Internalizing behavior problems$^{=}$; Externalizing behavior problems$^{=}$; Parent-child conflict$^{=}$**	**Parent-child conflict^{+}**	RCT	Internet resource condition
	Wade 2012 [175]							None	**Social problem-solving$^{=}$; Distress$^{=}$; Depression$^{=}$**		
Emotion regulation individual therapy for adolescents (ERITA)	Bjureberg 2018 [176]	Sweden	Pediatric / Other (Non-suicidal self-injury)	Parent / Y	1,2,7,9, 10	Entirely separate	CR only SP only	**Non-suicidal self-injury frequency^{+}**	Distress, punitive, and minimization reactions to child's expression of negative emotions; Support and encouragement of child's emotional expressions	Single-arm	N/A
EpApp	Le Marne 2018 [177]	Australia	Pediatric / Other (Epilepsy)	Parent / Y	2	Entirely separate	Shared CR only	**Knowledge acquisition^{+}**	Feedback on app functionality, design, content, and utility	Single-arm	N/A
Teens Taking Charge: Managing Arthritis Online	Stinson 2010 [178]	Canada	Pediatric / Other (Juvenile Ideopathic Arthritis)	Parent / Y	1,7,10	Entirely separate	Shared CR only SP only	**Health-related quality of life (QOL)$^{=}$**	None	RCT	Attention control
Self-Cathing Experience Journal (SC-EJ)	Holland 2015 [85]	USA	Adolescent-Young Adult / Other (clean intermittent catheterization)	Any / N	1	Not specified	Shared	Perceived safety of journal content; Satisfaction; Helpfulness; Impact on isolation and hope	Perceived safety of journal content; Satisfaction; Helpfulness; Impact on isolation and hope	Single-arm	N/A

Description			Population		Intervention			Outcomes		Study design & comparator	
Intervention Name	Study	Country	CR Age Group / Condition	SP Relationship / Required	System components	Dyad participation	Content target	CR Outcomes	SP Outcomes	Trial design	Comparator
Comprehensive Health Enhancement Support System (CHESS)	Chih 2013 [179]	USA	Adult / Cancer	Any / Y	1,10	Not specified	Shared, CR only SP only	None	**Physical burden$^{=}$; Preparedness$^{=}$; Negative mood^{-}**	RCT‡	CHESS + Clinician Report
	Gustafson 2017 [180]							**Proportion of improved threshold (severe) symptoms to all threshold symptoms^{+}; Proportion of threshold to all assessed symptoms^{+}**	None		
	DuBenske 2014 [181]							None	**Burden^{+}; Disruptiveness of caregiving to SP's daily routine$^{=}$; Negative mood^{+}**		Internet resources
	Gustafson 2013 [182]							**Symptom distress (as reported by SP)$^{+}$**	None		
Couplelinks	Fergus 2014 [183]	Canada	Adult / Cancer	Spouse / Y	1,7,9,10	Separate and together	Shared	Program satisfaction; Quality of facilitation; Website usability; Convenience	Program satisfaction; Quality of facilitation; Website usability; Convenience	Single-arm	N/A
	Ianakieva 2016 [184]	Canada	Adult / Cancer	Spouse / Y	1,7,9,10	Separate and together	Shared	Development of engagement model based on: Completion time; Accountability for delays in completion; Attitude towards the program	Development of engagement model based on: Completion time; Accountability for delays in completion; Attitude towards the program	RCT	UC
	Ianakieva 2019 [185]							Development of engagement model based on: Completion time; Accountability for delays in completion; Attitude towards the program	Development of engagement model based on: Completion time; Accountability for delays in completion; Attitude towards the program		
FOCUS	Northouse 2014 [111]	USA	Adult / Cancer	Any / Y	1,8,9	Entirely together	Shared	**Emotional distress^{+}; Health-related QOL^{+}**	**Emotional distress^{+}; Health-related QOL^{+}**	Single-arm	N/A
Healthy U, Healthy Us	Kim 2017* [186]	USA	Adult / Cancer	Any / Y	1,5	Entirely together	Shared	Enrollment; Retention; Acceptability	Enrollment; Retention; Acceptability	Single-arm	N/A
	Mazzer 2016* [187]							Enrollment; Retention; Satisfaction; Systolic blood pressure; Diastolic blood pressure; Servings of fruits and vegetables	Enrollment; Retention; Satisfaction; Systolic blood pressure; Diastolic blood pressure; Servings of fruits and vegetables		
Prostate Cancer Education and Resources for Couples (PERC)	Song 2015 [188]	USA	Adult / Cancer	Spouse / Y	1,7,8	Entirely together	Shared	Feasibility metrics (recruitment, retention, website activity data); Quality of life; Symptom distress; General symptoms; Dyadic communication; Relationship satisfaction	Feasibility metrics (recruitment, retention, website activity data); Quality of life; Distress related to partner's symptoms; General symptoms; Dyadic communication; Relationship satisfaction	Single-arm	N/A

Description			Population		Intervention			Outcomes		Study design & comparator	
Intervention Name	Study	Country	CR Age Group / Condition	SP Relationship / Required	System components	Dyad participation	Content target	CR Outcomes	SP Outcomes	Trial design	Comparator
Patient-centered healthcare decision making website	Vogel 2013 [189]	USA	Adult / Cancer	Any / N	1,10	Entirely separate	Shared, CR only SP only	**Completion of an advance healthcare directive$^{=}$; Participation in palliative care consultation$^{=}$**	None	RCT	Usual care informational resources by web
Web-based information tool	Bryant 2013* [190]	Australia	Adult / Cancer	Any / Y	1	Not specified	Shared	Perceived ease of use; Likelihood to share website with others; Access/use of tool	None	RCT	UC
Counseling About Regaining Erections and Sexual Satisfaction (CAREss) (WEB)	Schover 2012 [91]	USA	Adult / Cancer & other (sexual dysfunction)	Spouse / Y	1,7,10	Separate and together	Shared, CR only SP only	**Sexual function and satisfaction$^{+\text{UC},=\text{in-person}}$**	**Sexual function and satisfaction$^{=\text{UC},=\text{in-person}}$**	RCT	UC and in-person CAREss
Internet-based Cognitive Behavioral Therapy for Sexuality after Breast Cancer	Hummel 2017 [92]	Netherlands	Adult / Cancer & other (sexual dysfunction)	Spouse / N	1,7,8,9, 10	Separate and together	Shared, CR only SP only	**Sexual functioning^{+}; Intimacy$^{=}$**	None	RCT	UC
	Hummel 2018 [93]							None	**Secondary analysis of Tx arm only: Sexual functioning^{+}; Erectile function$^{=}$; Orgasmic functioning^{+}; Intercourse satisfaction^{+}; Sexual satisfaction^{+}; Emotional intimacy$^{=}$; Social intimacy$^{=}$; Sexual intimacy^{+}; Intellectual intimacy$^{=}$; Recreational intimacy$^{=}$; Conventionality$^{=}$**		
CarePartners (Diabetes)	Aikens 2014 [86]	USA	Adult / Diabetes	Any† / N	6,8,12	Entirely separate	CR only SP only	Attrition; engagement in Interactive Voice Response (IVR) calls; Problems reported during IVR calls; Clinician notifications	Participation	Patient-preference trial	With vs. without SP
	Aikens 2015a [89]							Physical health quality of life (QOL); Mental health QOL	None		
	Aikens 2015b [87]							Medication adherence; Physical health QOL; Mental health QOL	None		
FAMS (Family-Focused Add-On for Motivating Self-Care)	Mayberry 2016 [191]	USA	Adult / Diabetes	Any / N	7,8	Entirely separate	CR only SP only	User engagement; User experience	User experience	Patient-preference trial	N/A

Description			Population		Intervention			Outcomes		Study design & comparator	
Intervention Name	Study	Country	CR Age Group / Condition	SP Relationship / Required	System components	Dyad participation	Content target	CR Outcomes	SP Outcomes	Trial design	Comparator
MumMood Booster	Milgrom 2016 [192]	Australia	Adult / Mental health condition	Spouse / N	1,7,10	Entirely separate	CR only<br>SP only	**Changes in depressive diagnostic status^{+}; Depression symptom severity^{+}**	None	RCT	UC
CarePartners (Depression)	Aikens 2015c [88]	USA	Adult / Mental health condition	Any† / N	6,8	Entirely separate	CR only<br>SP only	**Antidepressant adherence^{+}; Depression remission^{+}; Bed days due to depression$^{=}$**	None	Patient-preference trial	with vs. without SP
SOAR (Schizophrenia Guide)	Rotondi 2005 [116]	USA	Adult / Mental health condition	Any / N	1,10,13	Separate and together	Shared<br>CR only<br>SP only	**Perceived stress^{+}; Perceived social support$^{=}$**	**Perceived stress$^{=}$; Perceived social support$^{=}$**	RCT	UC
	Rotondi 2010 [115]							**Changes in positive symptoms^{+}; Knowledge about schizophrenia^{+}**	**Knowledge about schizophrenia^{+}**		
	Rotondi 2013* [117]	USA	Adult / Mental health condition	Any / N	1,10,13	Separate and together	Shared<br>CR only<br>SP only	**Severity of positive symptoms^{+}; Knowledge of schizophrenia^{+}**	**Knowledge of schizophrenia^{+}**	RCT	UC
CarePartners (Chronic Heart Failure)	Piette 2015 [90]	USA	Adult / Other (Chronic Heart Failure)	Any† / Y	6,8	Separate and together	Shared,<br>CR only<br>SP only	Heart failure-specific QOL; Self-care	Caregiving strain; Depressive symptoms; Self-management support activities (caregiving time and activities)	RCT	Patient-focused mHealth service
F@ce	Kamwesiga 2018 [193]	Uganda	Adult / Other (Stroke)	Any / Y	7,8,13	Separate and together	Shared<br>CR only	**Performance of self-care^{+}; Satisfaction with self-care$^{=}$; Self-efficacy^{+}; Stroke impact$^{=}$**	None	RCT	UC
Internet-based Behavioral Couples Therapy	Nilsson 2017 [194]	Sweden	Adult / Other (Problem gambling)	Any / Y	1,8,9,10	Separate and together	CR only<br>SP only	**Gambling behavior$^{=}$; Problem gambling severity$^{=}$**	Depressive symptoms; Anxiety symptoms	RCT	Individual Internet-based CBT
Multifaceted web-based intervention	Dew 2004 [195]	USA	Adult / Other (post heart transplant)	Any / N	1,9,10	Not specified	Shared<br>CR only<br>SP only	Website accessibility and user satisfaction; Mental health; QOL; Medical compliance	Website accessibility and user satisfaction; Mental health; QOL	Single-arm	historical control

Note: CR= Care recipient; SP= Support person. RCT= Randomized controlled trial; UC= Usual care (includes waitlist control); Tx= Treatment; QOL=Quality of Life. “Parent” includes legal guardians; “Spouse” includes domestic/romantic partners.

*Conference abstract; **Other publication (i.e., dissertation, book chapter)

‡ DuBenske 2014 and Gustafson 2013 articles report data from a randomized clinical trial among people with lung cancer and their SPs (NCT00365963); Chih 2012 and Gustafson 2017 combine data from NCT00365963 with data from NCT00214162 which trialed the program among people with breast or prostate cancer and their SPs.

† CarePartners requires that the SP must not cohabitate with the PWI.

Merged cells across population, intervention, and study design columns indicate that records originate from the same trial data.

System components: 1= Browser-based components; 2= Mobile application; 3= Link with Electronic Medical Record; 4= Body-worn sensors; 5= Videoconferencing; 6= Automated telephone call; 7= Human telephone contact; 8= Automated emails/SMS; 9= Human emails/SMS; 10= Asynchronous communication portal; 11= Synchronous chat room; 12= CD-ROM/DVD; 13= In-person sessions

Bolded outcomes were specified by authors as primary outcomes; $^{+}$ indicates intervention significantly changed (single-arm) or superior to comparator (RCT) in hypothesized direction at post-intervention, $^{=}$ indicates no change (single arm) or intervention equivalent to comparator (RCT) at post-intervention, † indicates variables were correlated in hypothesized direction.
